# Effects of Xingnaojing on serum high-sensitivity C-reactive protein and neuron-specific enolase in patients with acute cerebral hemorrhage: A protocol of systematic review and meta-analysis

**DOI:** 10.1097/MD.0000000000021379

**Published:** 2020-11-06

**Authors:** Zhe-ren Zhou, Yun-hua Zhao, Rong Sun, Yu-rong Zhang

**Affiliations:** a Department of Emergency, Hospital of Xi’an Jiaotong University; b Department of Ultrasound; c Department of Emergency, Xi’an Gaoxin Hospital; d Department of Neurology, The First Affiliated Hospital of Xi’an Jiaotong University, Xi’an, China.

**Keywords:** acute cerebral hemorrhage, high-sensitivity C-reactive protein, neuron-specific enolase, xingnaojing

## Abstract

**Background:**

This study will systematically explore the effects of Xingnaojing (XNJ) on serum high-sensitivity C-reactive protein (hs-CRP) and neuron-specific enolase (NSE) in patients with acute cerebral hemorrhage (ACH).

**Methods:**

We will comprehensively search the following electronic databases (MEDLINE, EMBASE, Cochrane Library, Allied and Complementary Medicine Database, and China National Knowledge Infrastructure) from inception to the March 1, 2020. There are no limitations related to the language and publication status. Two authors will independently perform all citation identification, information extraction, and study quality. All potential conflicts will be solved through discussion with the help of a third author. RevMan 5.3 software will be used for data synthesis and statistical analysis.

**Results:**

This study will summarize the present evidence to investigate the effects of XNJ on serum hs-CRP and NSE in patients with ACH.

**Conclusion:**

This study may provide an impressive understanding of perspective from scientific basis for effects of XNJ on serum hs-CRP and NSE in patients with ACH.

**Study registration:**

PROSPERO CRD42020171648.

## 1 Introduction

Stroke occurs when the blood supply to the part of brain is interrupted or decreased.^[1,2]^ It is characterized by high incidence, disability, mortality, and recurrence rates.^[3–5]^ Among stroke conditions, acute cerebral hemorrhage (ACH) occurs as one of the highest incidence rates (varies from 50% to 80%) and disability rates (ranges from 50% to 70%).^[6,7]^ It accounts for 20% to 30% of all stroke disorders.^[8,9]^ Although a variety of treatments are available for ACH, they still suffer from satisfied results.^[10–14]^ Fortunately, traditional Chinese medicine has been utilized to treat stroke survivors effectively.^[15–19]^ Specifically, studies suggested that Xingnaojing (XNJ) has significant effects on serum high-sensitivity C-reactive protein (hs-CRP) and neuron-specific enolase (NSE) in patients with ACH.^[20–32]^ However, no systematic review has investigated the effects of XNJ on hs-CRP and NSE in patients with ACH. Thus, this systematic review aims to explore the effects of XNJ on hs-CRP and NSE in patients with ACH.

## 2 Methods and analysis

### 2.1 PROSPERO registration

This study protocol has been registered through PROSPERO (CRD42020171648). It is organized based on the Preferred Reporting Items for Systematic Reviews and Meta-Analysis (PRISRMA) Protocol statement.^[33]^

### 2.2 Study inclusion and exclusion criteria

#### 2.2.1 Types of studies

We will include potential randomized controlled trials (RCTs) investigating the effects of XNJ on serum hs-CRP and NSE in patients with ACH. We will not limit language and publication status to all studies.

#### 2.2.2 Types of interventions

All patients in the experimental group received XNJ as their management.

All participants in the control group underwent any interventions without restrictions. However, we will exclude studies involving any forms of XNJ.

#### 2.2.3 Types of participants

We will include all subjects who were diagnosed as ACH irrespective of religion, race, sex or place of birth.

#### 2.2.4 Types of outcome measurements

The primary outcomes include serum hs-CRP and NSE in patients with ACH.

The secondary outcomes are limb function (as measured by Fugl–Meyer Assessment scale, or related scales), muscle strength (as identified by the motricity index or connected tools), quality of life (as checked by activities of daily living scale or relevant scores), and adverse events.

### 2.3 Literature sources and search strategy

We will systematically search the electronic databases from inception to the March 1, 2020 in MEDLINE, EMBASE, Cochrane Library, Allied and Complementary Medicine Database, and China National Knowledge Infrastructure. No limitations will be applied to the language and publication status. The search strategy of MEDLINE is built (Table 1). We will also modify identical search strategies for other electronic databases.

**Table 1:** Search strategy for MEDLINE.

Number	Search terms
1	stroke
2	hemorrhagic stroke
3	acute cerebral hemorrhage
4	intracerebral hemorrhage
5	bleeding
6	brain hemorrhage
7	cerebral hemorrhage
8	blood supply
9	serum
10	high-sensitivity
11	C-reactive protein
12	neuron-specific enolase
13	Or 1–12
14	traditional Chinese medicine
15	herbal medicine
16	herbal formulas
17	Xingnaojing
18	injection
19	Or 14–18
20	randomized controlled trials
21	random
22	randomly
23	control
24	comparator
25	allocation
26	placebo
27	blind
28	clinical study
29	controlled study
30	Or 20–29
31	13 and 19 and 30

Besides, we will check other literature sources, such as conference proceedings, and reference lists of included studies.

### 2.4 Data collection and analysis

#### 2.4.1 Selection of studies

Two authors will independently check titles/abstracts of retrieved records and all unrelated studies will be removed. Full texts of potential studies will be identified in details and determined for eligibility. We will record all excluded studies with specific reasons. We will demonstrate process of study selection in a PRISMA diagram chart. Any different opinions between 2 authors will be resolved by a third author via discussion.

#### 2.4.2 Data extraction and management

A data extraction sheet will be designed previously to collect all essential information. Any conflicts between 2 authors will be settled down by a third author through discussion. The extracted information comprises of study characteristics (e.g., title, first author, and publication time), patient characteristics (e.g., age, sex, diagnostic criteria, and eligibility criteria), study setting, study methods, details of treatment and controls, outcome indicators, results, findings, and adverse events.

#### 2.4.3 Missing data dealing with

We will request any unclear or missing data from original authors by email if it occurs. If such data is not obtainable, we will analyze available data only using intention-to-treat analysis.

### 2.5 Study quality evaluation

Two authors will independently assess study quality using Cochrane risk of bias tool. This tool covers 7 domains and is further rated as low, unclear and high risk of bias for each item. Any disagreements will be solved by a third author through consultation.

### 2.6 Statistical analysis

RevMan 5.3 software will be utilized to analyze extracted data and to perform statistical analysis. We will estimate continuous data as weighted mean difference or standard mean difference and 95% confidence intervals (CIs), and will express dichotomous data as risk ratio and 95% CIs. We will use *I*^*2*^ statistic to test potential heterogeneity among included RCTs. *I*^*2*^ ≤ 50% means homogeneity, and we will use a fixed-effects model, and will perform a meta-analysis if it is possible. *I*^*2*^ > 50% suggests remarkable heterogeneity, and we will place a random-effects model, and will undertake a subgroup analysis to detect its possible sources. In addition, we will also conduct a narrative summary.

### 2.7 Additional analysis

#### 2.7.1 Subgroup analysis

A subgroup analysis will be carried out to investigate the sources of considerable heterogeneity based on the variations in study characteristics, study quality, interventions and controls, and outcomes.

#### 2.7.2 Sensitivity analysis

A sensitivity analysis will be explored to test the robustness of study findings by removing low quality study.

#### 2.7.3 Reporting bias

A reporting bias will be checked by funnel plot and Eggers regression test if we will include more than 10 eligible studies.^[34–35]^

### 2.8 Ethics and dissemination

This study will not need ethical document, since it will not obtain individual subject data. This study is expected to be published at a peer-reviewed journal.

## 3 Discussion

Previous studies have hypothesized that XNJ plays a key role in treating ACH. It has effects on hs-CRP and NSE in patients with ACH.^[20–32]^ However, all conclusions are drawn based on the individual study, and all evidence is still at the conceptual level. In addition, no systematic review specifically has addressed this issue. Therefore, this is the first study to systematically investigate the effects on hs-CRP and NSE in patients with ACH. The results of this study will provide beneficial evidence for both clinical practice and future studies.

## Author contributions

**Conceptualization:** Zhe-ren Zhou, Yun-hua Zhao, Rong Sun, Yu-rong Zhang.

**Data curation:** Zhe-ren Zhou, Rong Sun, Yu-rong Zhang.

**Formal analysis:** Zhe-ren Zhou, Yun-hua Zhao, Rong Sun.

**Funding acquisition:** Yu-rong Zhang.

**Investigation:** Yu-rong Zhang.

**Methodology:** Zhe-ren Zhou, Yun-hua Zhao, Rong Sun.

**Project administration:** Yu-rong Zhang.

**Resources:** Zhe-ren Zhou, Yun-hua Zhao, Rong Sun.

**Software:** Zhe-ren Zhou, Yun-hua Zhao.

**Supervision:** Yu-rong Zhang.

**Validation:** Zhe-ren Zhou, Yun-hua Zhao, Yu-rong Zhang.

**Visualization:** Zhe-ren Zhou, Yun-hua Zhao, Rong Sun, Yu-rong Zhang.

**Writing – original draft:** Zhe-ren Zhou, Rong Sun, Yu-rong Zhang.

**Writing – review & editing:** Zhe-ren Zhou, Yun-hua Zhao, Rong Sun, Yu-rong Zhang.
